# Physical condition and maintenance of mosquito bed nets in Kwale County, coastal Kenya

**DOI:** 10.1186/1475-2875-12-46

**Published:** 2013-02-01

**Authors:** Francis M Mutuku, Maureen Khambira, Donal Bisanzio, Peter Mungai, Isaac Mwanzo, Eric M Muchiri, Charles H King, Uriel Kitron

**Affiliations:** 1 Department of Environmental Studies, Emory University, Atlanta, Georgia; 2 Department of Public Health, School of Health Sciences, Kenyatta University, Nairobi, Kenya; 3 Center for Global Health and Diseases, Case Western Reserve University, Cleveland, Ohio, USA; 4 Division of Vector Borne and Neglected Tropical Diseases (DVBNTD), Ministry of Public Health and Sanitation, Nairobi, Kenya

## Abstract

**Background:**

Despite the extensive ownership and use of insecticide-treated nets (ITNs) over the last decade, the effective lifespan of these nets, especially their physical integrity, under true operational conditions is not well-understood. Usefulness of nets declines primarily due to physical damage or loss of insecticidal activity.

**Methods:**

A community based cross-sectional survey was used to determine the physical condition and to identify predictors of poor physical condition for bed nets owned by individuals from communities in Kwale County, coastal Kenya. A proportionate hole index (pHI) was used as a standard measure, and the cut-offs for an ‘effective net’ (offer substantial protection against mosquito bites) and ‘ineffective nets’ (offer little or no protection against mosquito bites) were determined (pHI ≤88 (about ≤500 cm^2^ of holes surface area) and pHI of >88 (≥500 cm^2^ of holes surface area), respectively).

**Results:**

The vast majority (78%) of the surveyed nets had some holes. The median pHI was 92 (range: 1–2,980). Overall, half of the nets were categorized as ‘effective nets’ or ‘serviceable nets’. Physical deterioration of nets was associated with higher use and washing frequency. Young children and older children were found to use ineffective bed nets significantly more often than infants, while the physical integrity of nets owned by pregnant women was similar to those owned by infants. Estuarine environment inhabitants owned nets with the worst physical condition, while nets owned by the coastal slope inhabitants were in fairly good physical condition. The results suggest that bed nets are optimally utilized when they are new and physically intact. Thereafter, bed net utilization decreases gradually with increasing physical deterioration, with most net owners withdrawing physically damaged nets from routine use.

This withdrawal commonly happens following 1.5 years of use, making bed net use the most important predictor of physical integrity. On average, the nets were washed twice within six months prior to the survey. Washing frequency was significantly influenced by the bed net colour and bed net age. Lack of knowledge on reasons for net retreatment and the retreatment procedure was evident, while net repair was minimal and did not seem to improve the physical condition of the nets. The “catch-up” bed net distribution strategies are sufficient for ensuring adequate ownership and utilization of ‘effective nets’ in the targeted groups, but bi-annual mass distribution is necessary to provide similar ownership and utilization for the other groups not targeted by “catch-up” strategies.

**Conclusions:**

Monitoring and maintenance strategies that will deliver locally appropriate education messages on net washing and repair will enhance the effectiveness of malaria control programmes, and further research to assess ineffective nets need is needed.

## Background

Long-lasting insecticide nets (LLINs) are now entrenched as a major anti-malarial intervention [1]. In most malaria-endemic countries > 50% of households own at least one insecticide-treated net (ITNs) [1]. Despite this extensive coverage and use of ITNs over the last decade, their effective lifespan, and especially their physical integrity, is not well-understood under true operational and varied epidemiological settings [2-6]. While there is standard method of quantifying the number of holes in a bed net, lack of a standardized method to define a functional bed net in the field is an important limitation of studies evaluating the effectiveness of bed nets [2].

Bed nets reduce human-vector contact by providing a physical barrier between the human sleeping under the bed net and the malaria vector mosquito. This protection is enhanced when the bed net is treated with an insecticide that deters, repels, or kills vectors that attempt to bite the sleeper. A bed net that has its mesh (the number of holes per square inch) intact will rarely allow mosquitoes to reach the person sleeping under the bed net [7]. However, with use bed nets accrue holes that are big enough to allow mosquitoes to pass through. Thus, a functional or useful net is one that is physically intact and has insecticidal protection [8]. In addition to evaluating the physical presence (retention) of nets, it is paramount for malaria control programmes to also investigate the ‘usefulness’ of surviving nets (nets under routine use) [8]. Recent publication of field guidelines on collection of hole data is commendable [9]. However, these guidelines provide only a standard approach of quantifying the number of holes on a net, but a measure of categorizing the holes in to ‘effective net’ (likely to fulfill their protective function) and ‘ineffective net’ (offering diminished or no protection from mosquito bites) is lacking [2].

Not surprisingly, most controlled experiments on the importance of holes on bed nets have concluded that whether treated or untreated, protection offered by bed nets diminishes with increasing number of holes [10-12]. In Tanzania, Olyset nets (LLINs) after seven years of use provided personal protection only when they were in good physical condition [13]. Reduced effectiveness of bed nets in poor physical condition has also been reported in different epidemiological settings. Untreated worn nets (≥5 holes of about 2 cm in diameter, or ≥5 holes of unknown in diameter) offered significantly reduced protection against malaria in rural Gambia and in Kilifi, Kenya [14,15]. In a recent study, untreated nets with holes were not protective, and untreated nets with no holes offered more protection against malaria infections than holed LLINs/ITNs [16]. Diminished protection from bed nets is further exacerbated if the vectors are resistant to the pyrethroids used in bed net [10,11]. Moreover, an increasing number of studies suggest that the frequency of bed net use decreases with increasing net age and increasing physical damage of nets [17-19].

Published studies on physical integrity of bed nets in Uganda suggest that considerable physical damage (45%-78% of damaged nets) can occur even within a year of bed net use in operational conditions [3,6]. Recently, Githinji and colleagues [20] reported poor physical condition in 40% of nets a year after distribution. Physical damage to nets is linearly correlated with reported bed net age and most bed nets are no longer in use beyond two years of use [2,5,6,21]. Under ‘real life’ conditions, LLINs are expected to have two to three years of useful life [22]. Nevertheless, various researchers have variably estimated the useful life of bed nets to be up to four years [4,5] As a result of the insecticidal effect on mosquitoes, insecticide treated nets have slightly longer useful life than untreated nets [4,5,23]. Regardless of insecticide treatment status, the rate at which holes appear on the net may be predisposed by net fabric [24] and fibre weight or denier [5]. Other factors that influence physical deterioration are the house environment (house wall material, bed type and construction), social economic status (SES) and the bed net maintenance behaviour (general handling, washing and repair) [6,9]. Animals (goats, sheep, cattle, rodents, cats), fire from various sources (oil lamps, sparks from cooking, candles) and snagging on the bed frame are some of the known causes of holes on bed nets [5,25].

Following the free mass distribution of bed nets in 2006, bed net ownership in Kwale County has been sustained at very high levels [26-28]. The distribution of free nets at maternal and child clinics to pregnant women and children under five years of age has been the primary approach through which most nets continuously get into the community. Secondary approaches include social marketing, where partial subsidy is offered, and *ad hoc* distribution by NGOs. Bed nets are also available through retail outlets at full cost for those who can afford them. Although the ownership situation continues to improve, the physical condition and maintenance behaviour of the nets in use is rarely evaluated and may undermine efforts to scale up ITNs. This paper describes the physical condition and identifies some of the predictors for poor physical condition of bed nets owned by individuals from communities in Kwale County, coastal Kenya. Additionally, net maintenance behaviour and presence and reported age of other nets that were not in routine use were documented.

## Methods

### Study area and population

**Figure 1:**
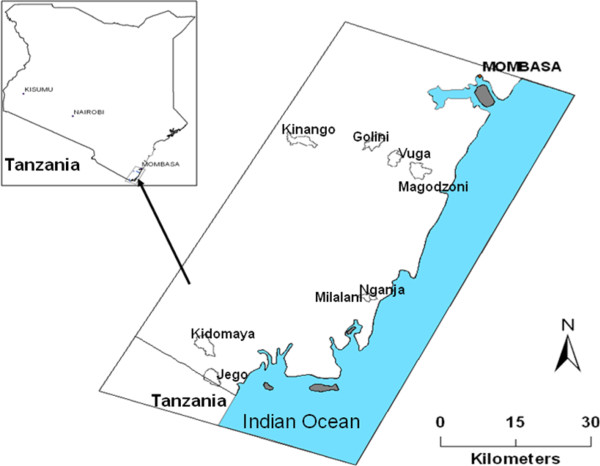
Map of the study area showing the study villages.

The survey was conducted in Msambweni, Vanga, Matuga and Kinango divisions of Kwale County, south coastal Kenya (4.03°S, 39.53°E, Figure 1). Kwale County borders Tanzania to the south-west and the Indian Ocean to the east. The area is hot and humid year round with annual mean temperatures range of 22°C - 34°C, average relative humidity range of 70% - 80%, and annual rainfall range of 900–1500 mm. Altitude ranges from 0 to 462 meters above sea level. Both malaria and lymphatic filariasis are endemic in the study area. The predominant vectors for human malaria are *Anopheles gambiae* s.l. and *Anopheles funestus* and they occur year-round with peaks of population abundance coinciding with seasonal rains [26,29].

Kwale County is mainly inhabited by Digo and Duruma communities with small proportions of Kambas and other communities, especially in urban areas, and a total population of 650,000 [30]. These are mainly subsistence farmers, growing cassava, cashew nuts, coconut, mangoes, and maize. Communities living further inland in Kinango maintain a substantial number of cattle, goats and sheep. Houses are constructed mostly of poles connected with sticks to create a frame for each wall that is then filled with mud, and supports the upper structure and the palm leaves that are typically used as roofing material.

### Study villages

The study villages were previously described by Mutuku and others [26]. Briefly, data was collected from eight villages grouped by four ecological settings defined by elevation, temperature, rainfall, relief, distance to the Indian Ocean and land cover type (Figure 1): 1) Jego and Kidomaya villages located at the southeastern tip of Kenya, representing the coastal estuarine environmental setting; 2) Nganja and Milalani villages located near Msambweni district hospital, representing the coastal plain setting; 3) Magodzoni village located at the bottom of the slope, Vuga village located at mid-slope and Golini village located at the top of the slope, representing the coastal slope setting); 4) Kinango located more inland, representing the inland semi-arid environmental setting.

### Ethical clearance

Ethical clearance was obtained by the Institutional Review Board at the University Hospital Case Medical Center of Cleveland, the Ethical Review Committee of the Kenya Medical Research Institute (KEMRI) and Emory University (Atlanta, GA).

### Household sampling

Households in the eight villages were previously mapped, with the number of households per village ranging from 175 to 513, with a total of 3,168 households. Sample size estimate was based on the proportion of households in malaria endemic areas in Kenya who had at least one insecticide-treated mosquito net (58%), according to the 2007 Kenya malaria indicator survey [31]. The standard error (SE) was assumed to be 7.5% at 95% confidence level. A sample size of 170 households per village was calculated. A total of 1360 randomly selected households in the study area were, therefore, targeted for administration of the structured questionnaire.

### Cross-sectional survey

A community based cross-sectional survey was conducted between December 2009 and April 2010 in the eight study villages using a structured questionnaire. The analysis reported here was performed only on those parts of the questionnaire focused on net attributes (colour, fabric, shape, size and brand), date of acquisition, whether purchased or not, and the exact location where the net was acquired. The total number of people who slept under the net the night prior to the survey and their person-types was also recorded. Additional information collected included reported frequency of washing, insecticide retreatment, and observed number of repairs and total count of all observed holes. Location of holes was recorded and categorized as on the head, foot, back, front or top sides of the net. In the survey, holes were categorized as small (smaller than an old KES coin - 2.7cm in diameter) or large holes (larger than this coin). The questionnaires were administered by community health workers who were familiar with the study area, the local language and the culture of the study community.

### Data analysis

The current net distribution strategies favor vulnerable groups (pregnant women and infants), and therefore the age and condition of the bed net depend on who owns or uses the bed net. Individuals owning or using bed nets were divided into five demographic categories of “person-type” by age and vulnerability status: infants (0–1 year), young children (2–5 years), older children (6–18 years), and adults (>18 years). The two holes sizes were aggregated into a proportionate holes index (pHI) by applying the following formula: pHI = ((small holes) + (Big holes*18)). The multiplication factor was chosen to reflect the approximate surface areas of the hole sizes (5.7 and 103.0 cm^2^, respectively) resulting in one unit of the pHI being equivalent to the area of KES coin (5.7 cm^2^) of hole surface [2,9]. Small holes were not weighted because they tended to be smaller or equal in size to the KES coin while most large holes were much bigger than the KES coin. The approximated diameter range of the large holes was 36cm, hence the multiplication factor of 18 (the median diameter).

Using the pHI, bed nets were categorized as ‘effective nets’ (offer substantial protection against mosquito bites) or as ‘ineffective nets’ (offer little or no protection against mosquito bites). Nets with pHI ≤ 88 (approximately ≤ 500 cm^2^ of holes surface area) were considered ‘effective nets’ and having a pHI of > 88 were considered ‘ineffective nets’. The high threshold used here for ‘effective nets’ (≤500 cm^2^) was deemed appropriate because most holes (about 50%) occurred at 10–30 cm from the bottom of the net ([6,25], personal observation: FM); an area that is usually tucked under the mattress when the bed net is in use.

Logistic regression using a generalized additive model (GAM) was applied to assess the predictors of bed net physical integrity. Physical condition of the net (1 = ‘effective net’ or 0 = ‘ineffective net’) was the model outcome variable. The independent variables included in the model were: net age, net colour, net fabric, net shape, net size, frequency of use, environmental or ecological setting, number of people (net users) per net, SES, demographic category and frequency of washing during the last six months prior to the survey. Before the logistic model was performed, all response variables of interest were subjected to univariate analysis using Kruskal-Wallis test. Multivariate Kruskal-Wallis analysis based on Mann–Whitney test with Bonferroni correction was used to compare net age among groups (demographic categories and ecological setting). Age of the bed net was included as nonlinear predictor. The best GAM logistic model (among all tested models) was chosen using a multi-model selection. This selection methodology compared a set of candidate models with each other and identified the best model (or the best set of models) based on model fit [32]. The best model was the one with the lowest Akaike information criterion (AIC) value. When the lowest AIC value differed from the next best model by < 2 units, a best set of models rather than a single best model was identified. The Akaike weight (ωi) for each model was calculated to evaluate the probability that a particular model fitted the data better than the alternative set of candidate models [32].

## Results

The survey included interviews with household heads and inspection of bed nets in 1176 households with 5526 individuals and 2,786 nets. Of the 2,786 nets, 1,849 (66%) were in routine use while 937 (34%) were not. Eighty percent (942/1176) of the households owned at least one net that was routinely used. Nets under routine use were further categorized as effective or ineffective nets. Overall, despite 82% of the total people who slept in the households surveyed having had any net and over 96% of nets found hanging, only 63% of the people slept under a net the night prior to the survey. The 937 nets not under routine use were identified in 49% (576/1176) of the households surveyed. These were further categorized as misused nets, physically damaged nets, extra nets or acceptability nets.

### Characteristics bed nets in routine use

Table 1 is a summary of characteristic of the bed nets owned by study participants. The dominant source of the bed nets was the government through public hospitals (63%) and retail outlets (23%), with NGOs and other sources accounting for 11%. By distribution channels, the Ministry of Health (MOH) in conjunction with population services international (PSI) and UNICEF distributed 71% of the bed nets (57% by PSI and 14% by UNICEF). Other distribution channels accounted for 13% of the nets, and distribution channels for 16% of the nets were unidentified. Of the 1,173 bed nets that household heads indicated were acquired from the hospitals, the majority (78%, 917/1173) were provided at no cost, 18% were purchased at a cost of ≤50 KES and only 4% were acquired at a cost of ≥ 100 KES. Of the bed nets acquired from retail outlets, 82% were bought at ≥100 KES (range: 100–950) with 9% costing ≤50 KES and the cost of the rest unknown. By bed net brands, the majority (68.0%) of the nets were ‘Olyset nets’, followed by ‘PermaNet nets’ (7.8%) and ‘Supanet nets’ (4.2%). Other brands (total of 5.3%) included ‘Supanet net extra’, Safinet, and ‘Sunflag Mmbu net’; the brands of 14.7% of the nets could not be verified. Overall, most net characteristics including colour, shape, fabric and size were dependent on the two most common net brands; Olyset and PermaNet. Olyset nets were predominantly blue (78%) or white (21%) in colour, and large (77%) or of unknown size (16%), while PermaNet nets were mostly white (60%), blue (27%) or green (13%) in colour, and either large (53%), extra large (30%) or medium (13%). The fabric of all Olyset nets was polyethylene, and for all other nets it was polyester.

**Table 1:** Characteristics of bed nets owned during the cross-sectional survey (December 2009-April 2010)

**Variable**	**N = 1849**	**% bed nets**
Age in years*		
0	69	3.7
1	567	30.7
2	361	19.5
3	222	12.0
4	337	18.2
≥5	197	10.7
Don’t know	96	5.2
**Colour**		
Blue	1227	66.4
Green	141	7.6
White	481	26.0
**Shape**		
Rectangle	1499	81.1
Round	348	18.8
**Fabric**		
Polyethylene	1255	67.9
Polyester	594	32.1
**Size**		
Extra large	64	3.5
Large	1429	77.3
Medium	24	1.3
Small	41	2.2
Don’t know	291	15.7
**Source**		
Government	1173	63.4
Retail	422	22.8
Others	200	10.8
Don’t know	54	3.0
**Cost**		
Free	1100	59.5
≤ 50 KES	250	13.5
≥100 KES	399	21.6
Don’t know	100	5.4
**Brand**		
Olyset (LLIN)	1257	68.0
PermaNet (LLIN)	144	7.8
Supanet extra (LLIN)	21	1.1
Others	155	8.4
Don’t know	272	14.7
**Frequency of use**		
0-2 times/week	162	8.8
3-4 times/week	53	2.9
5-7 times/week	1620	88.3

*Nets acquired in 2010 were assigned age 0 because the survey ended in April, 2010.

### Washing and retreatment of bed nets in routine use

Data on frequency of bed net washing in the last six months prior to the survey were available for 1,809 bed nets: 19% had not been washed at all, 52% had been washed either once or twice (the recommended washing frequency) and 29% were washed at least three times. On average, the nets were washed 2 times (range: 0–30 within six months prior to the survey. Washing frequency was significantly influenced by the bed net colour (Kruskal-Wallis χ^2^ = 44.04, DF = 2, *p* < 0.0001), with blue-coloured nets the least often washed (mean: 1.9); similar number of washes were reported for green (mean: 2.8) and white coloured nets (mean: 2.5). Bed net washing frequency also differed significantly with bed net age (Kruskal-Wallis χ^2^ = 99.91, DF = 5, *p* < 0.0001), with newer bed nets (≤1 year) washed less often than older nets (>1 year).

Respondents reported re-treating 33% of nets (n = 1,839). Lack of knowledge as to which nets should be re-treated emerged as an important issue: the majority (69%, 426/616) of the nets reported to have been ever re-treated were LLINs. Of the Non-LLINs, 44% were re-treated within periods ranging from < 6 months to >9 months. Almost all (98%) of the re-treatment was done with deltamethrin (branded as Power Tab); 0.2% used alpha-cypermethrin (branded as Fedona) and 1.8% did not know the insecticide used. The cost for 71% of the re-treatment kits was KES 10–80 while 21% were free. Further lack of knowledge on reasons for re-treating bed nets and the re-treatment procedure was exhibited by the responses about the reasons for non-treatment of the bed nets (Table 2). While cost of the re-treatment insecticide was the most important reason accounting for 49% of the responses, lack of access to the insecticide and know-how of the re-treatment procedure featured prominently, accounting for ~25% of the reasons (Table 2).

**Table 2:** Reasons for non-retreatment of bed nets

**Reasons for not retreating**	**N (%)**
Cost of re-treatment insecticide	576 (49)
Access to the insecticide (not being available in retail shops)	154 (13)
Bed net is new	133 (11)
Lack of knowledge on how to do the re-treatment	130 (11)
Bed net is an LLIN	82 (7)
Fear of the effects of retreatment insecticide (irritates, allergic)	39 (4)
Others (bed net is old, is rarely used, there are no mosquitoes, being busy)	60 (5)
**Total**	**1,174 (100)**

### Physical condition of bed nets in routine use

Of the 1,849 nets surveyed, 1,843 were inspected for presence of holes. Out of the 1,843 bed nets, 1,436 (78%; 95% CI: 76% - 80%) had holes or were torn. In total, 53,932 holes were counted; 66% of which were small holes. The median pHI was 92 (range: 1–2,980). Overall, half of the nets (932/1,843; 95% CI: 48% - 53%) were categorized as ‘effective nets’ or serviceable nets. Respondents indicated that most holes (56%) were caused by the bed frame or mattress during tucking-in of the net. This was in line with the finding that most holes (31%) were located on the front side of the net; the side that is always used, especially for nets that remain in position over the sleeping place (hanging). The back side (23%), foot side (22%) and head side (21%) had almost equal proportions of holes while the top side (3%) had the least number of holes. A high proportion of the holes were also caused by fire; mostly in single roomed houses where the room serves as both kitchen and bedroom. Another source of fire are the tin lamps, that are used by >70% of the families. Holes are also caused by domestic animals (mostly goats and sheep), especially when people share same sleeping spaces with these animals, and by rats and other rodents (Table 3). Other causes for holes that were mentioned included toe nails, over-washing, laundry hanging lines, kids playing and tears that occur when the net comes in conduct with the house wall. Net repairs were noted in 21% (308/1,436) of the nets found with holes in 228 households. Repairs were done primarily by the female household head and rarely by another member of the household. There was an average of five repairs per net among the repaired nets (Range: 1–35). Repairs were mostly done in households either without any other net (42%; 97/228) or with only one other net (31%; 71/228). Repairing the nets did not seem to improve their physical integrity, given that most of the repaired nets (95%) were full of holes (high pHI) and hence were categorized as ‘ineffective nets’.

**Table 3:** Causes of holes in bed nets

**Causes**	**N**	**(%)**
**Bedframe and mattress**	802	56
**Animals**	152	11
**Fire**	150	10
**Age of Net**	131	9
**Others**	43	3
**Don’t know**	158	11
**Total**	**1,436**	**100**

### Relationship between bed net age; person-type and physical condition of nets in routine use

**Figure 2:**
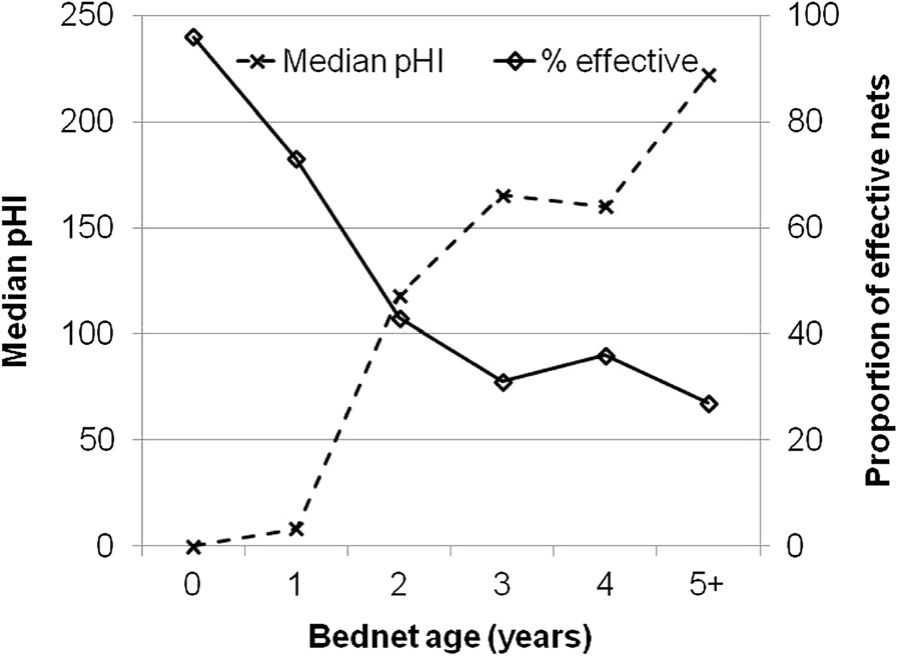
Median proportionate hole index (pHI) and proportion of effective bed nets by bed net age (as reported by respondents).

Respondents reported knowing the age of 95% (1,753/1,849) of all bed nets. The reported mean age of the nets was 2.4 years (SE: 1.5, Range 0–10, Median: 2). Proportions of bed nets age used by different person-types are shown in Table 4. The number of bed nets used decreased with increasing bed net age for bed net 0–3 year old. The impact of the 2006 mass distribution of bed nets was evident; there were slightly more 4 year old bed nets than 3 year old ones. The rate of physical deterioration increased linearly with increasing bed net age (Figure 2).

**Table 4:** Proportions of bed nets owned by different demographic categories (as reported by respondents)

**Demographic category**	**≤1y (%)**	**2y (%)**	**3y (%)**	**4y (%)**	**>4y (%)**	**Mean age (SE)**
Pregnant women	48.5	9.1	15.1	18.2	9.1	2.2 (1.6)
Infant (0-1yrs)	67.1	11.8	9.2	7.9	4.0	1.6 (1.3)
Young children (2-5yrs)	39.8	20.8	14.4	17.6	7.4	2.3 (1.4)
Older children (6-18yrs)	30.6	21.6	11.5	23.9	12.4	2.6 (1.5)
Others (above 18yrs)	39.0	21.0	12.9	17.0	10.1	2.0 (1.5)
**Total**	**36.3**	**20.6**	**12.7**	**19.2**	**11.2**	**2.4 (1.5)**

**Figure 3:**
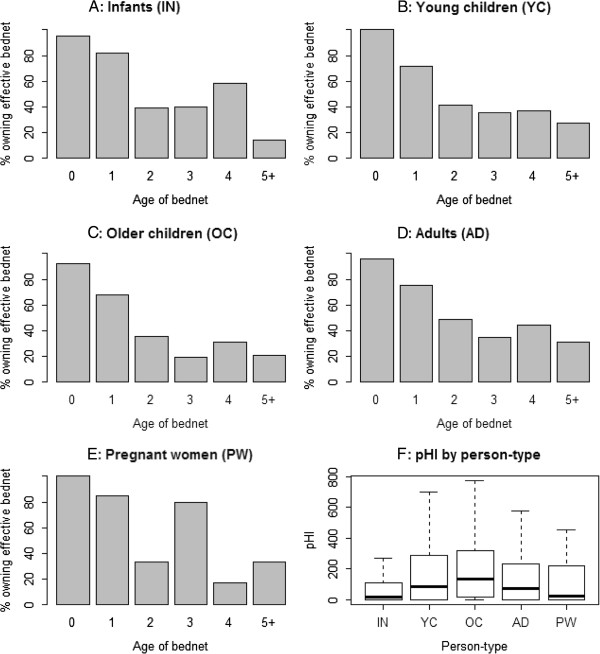
Proportion of people by demographic category that owned effective bed nets as a function of bed net age (A-E) and a box plot for pHI (F) In-Infants; YC-Young children; OC-Older children; Ad-Adults, PW-Pregnant women.

The age of the bed nets varied significantly with the person-type (Kruskal-Wallis χ^2^ = 75.76, p < 0.0001). Post-hoc analysis showed that the age of bed nets owned by young children, adults and pregnant women was comparable (Table 4). Infants owned the newest bed nets compared to all other person-types, while adults owned newer bed nets than older children. Consequently, the physical condition of bed nets varied significantly between different demographic categories (Kruskal-Wallis χ^2^ = 65.73, *p* < 0.0001). Bed nets used by infants and pregnant women were in the best physical condition (Figure 3F). Young children used bed nets in better condition compared to older children and adults, but the difference with adults was just above the 0.05 significant level (χ^2^ = 3.82, *p* < 0.06). Bed nets used by older children were in the worst physical condition, while adult bed nets were only better relative to those owned by older children (Figure 3F). Accordingly, not only did the proportion of people protected by ‘effective nets’ vary with bed net age but also with demographic category (Figure 3A-E). Among pregnant women, infants, young children, older children and adults, 62%, 71%, 50%, 41% and 55% owned ‘effective nets’ respectively.

Among those who owned ≤1year old bed nets, the proportion of people protected by ‘effective nets’ was 92%, 88%, 86%, 80%, and 86% for pregnant women, infants, young children, older children and adults respectively (Figure 3A-E). This trend held across the other bed net ages (Figure 3A-E). For bed nets ≥2 years old, the proportion of people protected by ‘effective nets’ was 41%, 38%, 35%, 26% and 40% for pregnant women, infants, young children, older children and adults respectively

### Environmental setting for bed nets in routine use

The age of bed net used in each environmental setting differed significantly (Kruskal-Wallis = 54.93, *p* < 0.0001).The age of bed nets in years in the coastal plain (mean: 2.81) and estuarine (mean: 2.88) environments were similar (p > 0.5) but significantly different from the coastal slope (mean: 2.33) and semi-arid inland (mean: 2.10) environments (p < 0.0001). Bed net age was not statistically different between bed nets found in the coastal slope and semi-arid inland environments (p > 0.05). The physical condition of the bed nets in the different environmental settings did not correspond to their ages.

**Figure 4:**
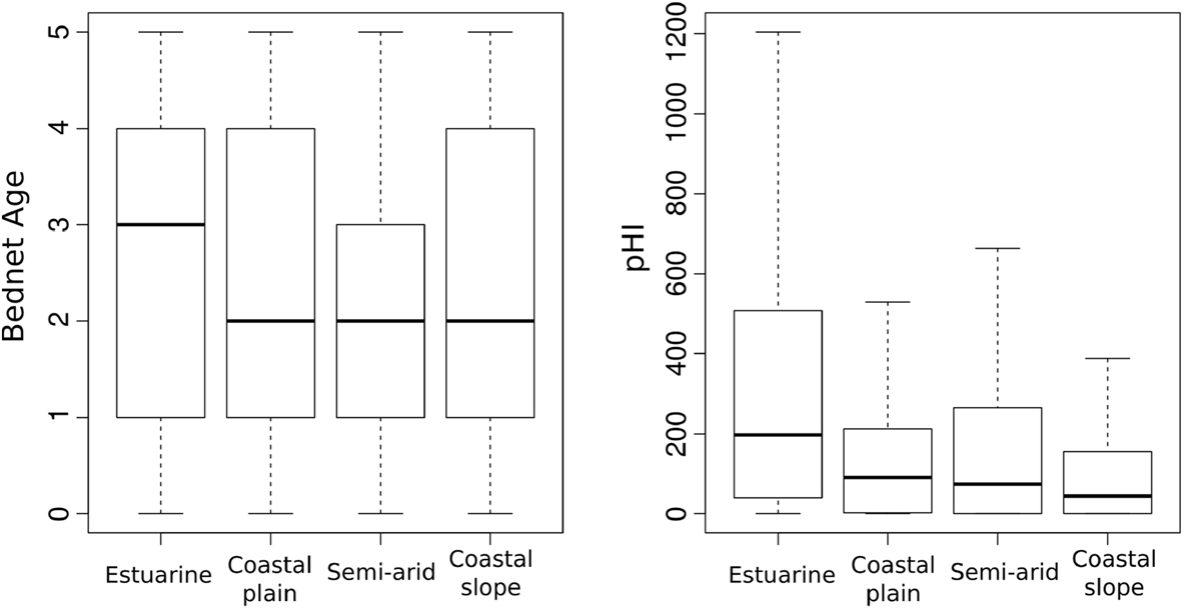
Bed net age and PHI by environmental setting.

As would be expected, bed nets in the estuarine environment were in worse physical condition; followed by semi-arid inland, and coastal plain while the coastal slope had the least damaged bed nets (Figures 1 and 4). In the coastal slope, coastal plain and semi-arid inland the frequency of bed net use (5–7 nights/week) was 77%, 90% and 95% respectively, suggesting sustained use resulting in faster physical deterioration. However, this observation did not hold for the estuarine environment where bed net use frequency was 93%, indicating that other factors contributed to bed net poor physical condition.

### SES, bed net shape and fabric of bed nets in routine use

Predictors of bed net physical condition that did not make it in the best model, but were significant in univariate analysis included SES, bed net shape and fabric. Poverty influenced nets’ physical condition considerably; nets from higher SES families (families in 4^th^ and 5^th^ quintiles) were in significantly better physical condition compared to those from lower SES families (families in quintiles 1–3). Compared to round bed nets, rectangular bed nets were in better physical condition (χ^2^ = 18.71, *p* < 0.0001), meaning that Olyset nets, that were nearly always (99.7%) rectangular are more durable compared to other net brands (Additional file 1: Additional material S1). The influence of net fabric was confounded by the fact that polyester nets were fewer and older compared to polyethylene nets. Similarly, round nets were fewer and older compared to rectangular nets.

### Predictors of physical condition of bed nets in routine use

**Figure 5:**
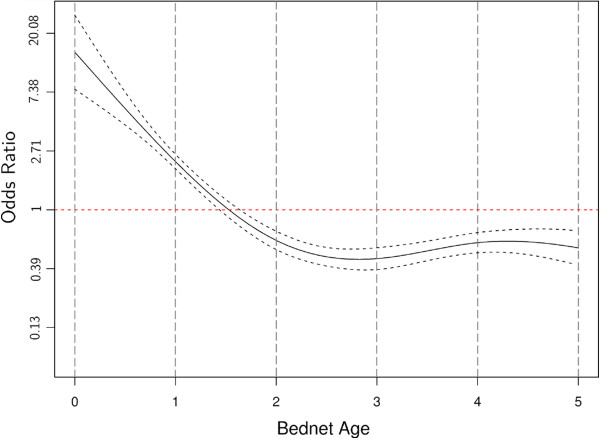
**Bed net age Odds ratio as a nonlinear predictor of an effective net.** The image includes the mean value and the 95% CI.

Results of the best GAM logistic model are presented in Table 5. The first eight models with a ωi >0.01 are shown in Table 6. The best model (model number one; with the lowest AIC value and highest ωi) included as predictors the age of the bed net, the washing and use frequency, the person type, the net color, and the environmental setting. Physical deterioration of nets occurred with increasing bed net washing frequency (OR = 0.89, 95% CI: 0.81-0.93). Physical deterioration of bed nets was positively associated with increasing frequency of use but this association was not significant for bed net use of 3–4 nights or 5–7 nights per week in comparison to 1–2 nights. Relative to infants, young children and older children were found to use the ineffective bed nets significantly more often, but the physical integrity of nets owned by pregnant women was similar to those owned by infants (Table 5). People in the estuarine environment owned nets in the worst physical condition while those in the coastal slope owned bed nets in fairly good physical condition. As a result of the distribution strategies (most nets were of same brand and sourced through the maternal and child clinics distribution), most nets were similar in terms of net fabric, colour, size, brand and shape; with univariate analysis showing similar trend in their association with pHI. Thus, only net colour was included in logistic regression. Different from other predictors, the age of the bed net was included in the GAM model as a non-linear predictor. It thus possible to calculate the odds of an ‘effective net’ after every year of net use. The odds ratio of the age remained positive from 0 to 1½ years and reached the peak of negative value in the half of the second year of use (Figure 5). Other than a slight increase during the 4^th^ year of bed net use due to free mass distribution that was done four years prior to this study, the odds ratio of the age remained almost constant from 3^rd^ year onwards.

**Table 5:** Linear predictors of the best GAM model for physical integrity of bed nets in coastal Kenya

**Predictors**	**Odds ratio (95% CI)**
Bed net washing frequency	0.89 (0.81-0.93)*
*Person-type (Ref: Infants)*	
Young children (2-5yrs)	0.54 (0.34-0.87)*
Older children (6-18yrs)	0.42 (0.30-0.69)**
Adults (above 18yrs)	0.64 (0.43-1.03)
Pregnant women	1.05 (0.44-2.36)
*Bed net use frequency (Ref: 1–2 nights/week)*	
3-4 nights/week	0.57 (0.28-1.08)
5-7 nights/week	1.15 (0.71-1.58)
*Net colour (Ref: White)*	
Green	0.81 (0.55-1.20)
Blue	1.60 (1.23-1.89)**
*Environmental setting (Ref: Estuarine)*	
Coastal plain	**2.31 (1.84-3.01)**
Coastal slope	3.45 (2.40-4.16)**
Semi-arid inland	2.10 (1.39-2.57)**

Significant predictors of an ‘effective net’ are in bold (* = p < 0.05, ** = p < 0.01)

The odds ratio represents the probability of owning an ‘effective bed net’.

**Table 6:** Akaike weight for the GLM logistic model with weight > 0.001

**Model number**	**Age**	**Person type**	**Bed net colour**	**SES**	**Ecological setting**	**Frequency of use**	**N people**	**Washing frequency**	**df**	**logLik**	**AICc**	**delta**	**ωi**
1	+	+	+	-	+	+	-	+	15	−1429.68	2889.55	0	0.52
2	+	+	+	-	+	+	-	+	14	−1431.94	2892.05	2.50	0.15
3	+	+	+	-	+	+	+	-	14	−1431.95	2892.08	2.52	0.14
4	+	+	+	-	+	+	-	-	13	−1433.95	2894.05	4.49	0.05
5	+	+	+	+	+	+	+	+	19	−1428.19	2894.70	5.15	0.04
6	+	+	+	-	+	-	+	+	13	−1434.55	2895.24	5.68	0.03
7	+	+	+	+	+	+	-	+	18	−1430.52	2897.32	7.77	0.01
8	+	+	+	+	+	+	+	-	18	−1430.55	2897.38	7.83	0.01

### Bed nets not under routine use

A total of 937 other nets that were not routinely in use for protection against mosquitoes, but were found within the houses, were surveyed. These nets were categorized into four groups: i) misused nets (28%) - nets that at the time of the survey were observed to be under another use other than protection against mosquito bites (Table 7) ii) physically damaged nets (34%) - nets with physical damage and were thus considered useless but had not been completely discarded from the house, iii) Extra nets (25%) - were mostly intact nets but were not being used at the time of the survey and the respondents claimed they were ‘extra nets’ (they are not being used because all household members have sufficient nets) iv) Acceptability nets (13%) - nets that were not in routine use because of acceptability related reasons. These reasons included the net being preserved for visitors, either too small or large for the bed, burnt, too hot and/or no mosquitoes.

**Table 7:** Observed bed net misuse

**Bed net misuse**	**N (%)**	**% ≤ 2yrs old**
Bed net were observed to be the material or part of material for:		
Chicken shelter	118 (44)	20
Screening windows	58 (22)	22
Bathing shelter	32 (12)	9
Nursery bed	17 (6)	0
House wall material	11 (4)	27
Fences (reinforces palm leaves fences)	11 (4)	36
Fishing	6 (2)	50
Door curtain or room divider	5 (2)	40
As beddings and/or mattress	4 (2)	0
Others	4 (2)	25
**Total**	**266** (100)	**21**

The reported mean age of the ‘damaged nets’ was 4.4 years (Range 0–8, Median: 4) and the majority of them had been distributed in 2006 during the free mass distribution. Most net misuse was observed in their use in chicken coops to restrict movement of the very young chicks or to restrain the whole brood of chicken from wandering into crop fields. Screening house windows to prevent mosquito entry and in construction of outdoor bathing shelters were the other common observed bed net misuses (Table 7). The reported mean age of the ‘misused nets’ was 4.0 years (Range 0–14, Median: 4, and the majority (79%) were >2 years old with observed moderate to severe physical damage. In fact, the age profile for ‘misused nets’ was similar that of ‘damaged nets’ and that of ‘extra nets’ was comparable to that of ‘acceptability nets’. Likely the ‘misused nets’ were worn-out nets or damaged nets that are no longer in use for protection against mosquitoes and thus only a small proportion (21%) of the reported misused nets constituted actual net misuse (nets that were ≤ 2 years old). Therefore the actual misuse of nets was minimal and was most common for fishing, door curtains and fences (Table 7). The reported ‘extra nets’ were relatively newer nets with a reported mean age of 2.0 years (Range 0–9, Median: 1); 70% of the ‘extra nets’ were ≤2 years old. The definite extent of ‘extra nets’ was also overestimated; slightly over a half (90/171; 53%) of the houses reporting presence of ‘extra nets’ did not have sufficient bed nets for all household members.

## Discussion

In this study, the physical condition of bed nets of various ages as reported by net owners in a malaria endemic region in coastal Kenya was examined. At least one hole was observed in more than 3/4 of the bed nets surveyed regardless of age, and overall a half of the bed nets were categorized as ‘ineffective’ based on criteria of pHI developed here. The rapidity with which bed nets deteriorated physically was such that less than half of the bed nets were ‘effective’ following two years of use. The results suggest that bed nets are optimally utilized when they are new and physically intact. Bed net utilization decreases gradually with increasing physical deterioration, and most net owners withdraw physically damaged nets from routine use usually after 1.5 years of use [33]. The premise behind consistent use of bed nets during the first year of acquisition and progressively reduced consistency of use in subsequent years is based on their perceived usefulness [18,34]. Besides being perceived as having diminished mosquito-repelling properties [34], torn nets are also perceived as unlikely to act as a barrier for host-seeking mosquitoes. Thus, the rapid decline in physical condition is almost entirely a function of regular and consistent use, and net owners likely do not find the nets of much use following 1.5 years of use. The finding that most of the ‘effective nets’ were less than two years old and that most of nets not in routine use were more than two years old further support this observation. As demonstrated by Ngufor and others [11], non-use of even the slightly torn nets is likely to become more widespread given growing insecticide resistance of mosquitoes [35]; further diminishing the effectiveness of bed nets. These data imply that net users do not perceive them as protective following two years of use, and it is thus recommended that programmes aim to replace nets on a bi-annual basis.

The absence of a consistent method to measure the physical integrity of bed nets hampers effective comparisons of the findings of this study with those of previous studies. However, a consensus is emerging that under operational conditions physical deterioration significantly shortens bed nets’ effective lifespan from the previously estimated 2–4 years [3-5,7] to 1.5-2.5 years. Moreover, recent accounts of high proportions of damaged nets within a year of distribution [3,6,17,19,20] concur with findings that most of nets are physically damaged by 1.5 years of use. Additionally, 1/3 of nets found discarded in a study in Ethiopia were 12–24 months old [34]. These observations help explain the occurrence of substantial number of ‘damaged’ and ‘misused’ nets within the houses surveyed. The majority of nets reported as ‘misused nets’ were actually physically damaged nets. Deployment of these nets to uses other than protection against mosquitoes did not therefore constitute net misuse; the extent of bed net misuse was likely overestimated. This finding re-affirms a recent opinion that bed net misuse is not as widespread as earlier portrayed [36]. Besides physical damage, socio-cultural and environmental factors contributed to withdrawal of nets from routine use (non-utilization) resulting in ‘extra’ and ‘acceptability’ nets. Existence of undamaged nets that are no longer in regular use because they are purportedly ‘extra nets’ or ‘acceptability nets’ points to persistence of some of the barriers to use of bed nets [28]. Intensive information campaigns targeting all individual net users would help lower these barriers.

It is expected that higher bed net use frequency will result in poor physical integrity. The association between reported bed net use and physical condition though positive, was not as strong as anticipated. This inconsistency is likely a consequence of reliance on self-reports to ascribe bed net use. Self-reported bed net use is unreliable because it often misrepresents regular bed net use. Several studies have demonstrated that perceived or actual mosquito density is the main motivation for using bed nets [37-40], and that, therefore, mosquito densities could indirectly be used to predict actual bed net use. In recent mosquito surveys, a general trend of decreasing vector densities with increasing altitude in the study area was observed (Mutuku FM, unpublished data). In three of the four ecological settings with an altitude range of 4–238 meters, the highest proportion of malaria vectors (78%) were collected in villages (Jego and Kidomaya) located at lowest altitude whereas 18% and 4% of the vectors were collected in villages located at the mid (Milalani and Nganja) and highest (Kinango) altitudes respectively. A similar trend in mosquito numbers was observed within the coastal slope villages; where 56%, 38% and 6% of the vectors were collected from the village located at the bottom (Magodzoni), mid (Vuga) and top (Golini) of the slope, respectively. The emerging pattern in the study area of declining mosquito densities with increasing altitude had previously been reported in several other ecological settings [41-43]. Understandably, bed net use decreased with increasing altitude as did the physical deterioration of the nets. Thus, the differences in bed net physical deterioration in the different ecological settings are likely explained by variations in actual bed net use rather than the reported use.

Before ITNs became widely adopted as the primary malaria prevention tool, published data had demonstrated that physically damaged untreated nets were less effective or completely useless in protecting people sleeping under them [44-46]. On the other hand, significantly low protection was offered by torn insecticide treated bed nets; the degree of protection was reported to decrease with increasing physical damage and decreasing insecticide content [4,5]. Arguably, the low uptake of bed nets then and the perception that torn insecticide treated nets were still effective did not provide the requisite impetus to study the importance of physical damage on effectiveness of insecticide treated nets. However, the recent massive bed net scale-up efforts across Africa and the subsequent adoption of bed nets as the primary malaria prevention tool have created demand for evaluative studies. In addition to reporting impressive bed nets ownership with some countries reaching saturation levels, all these evaluative studies have also reported at least some aspect of physical condition of the nets [11,16-19,25,47,48]. Despite this current focus, standard procedures for measuring bed net physical integrity are still deficient [2]. Research to elucidate socioeconomic and cultural importance of damaged nets is necessary. How do net owners define a worn-out net or a useless net in as far as protecting oneself against mosquitoes is concerned? How are worn-out nets disposed off and are there uses for worn-out nets? Answers to these questions will provide valuable insight for assessing the useful life span of bed nets.

Differences in net use by demographic category are quite common in many countries in Africa, where higher proportions of pregnant women and infants and in some cases young children (< 6 years) are reported to use nets more commonly than older children (6–18 years old) and other adults [19,21,33,49,50]. Thus, the finding that bed nets in better physical condition were more likely to be used by infants and pregnant women was not surprising, because the nets are largely acquired free of charge. However, not only are the younger and older children unlikely to use any net but they also use nets of poor physical quality; an observation also reported from western Kenya [20] and Tanzania [50]. These variations in net use and fabric integrity among the different users are mainly attributable to net distribution strategies. It is likely that the vulnerable groups (pregnant women and infants) pass on physically damaged nets to the other non-targeted family members (other adults and older children) [51]. Thus, the well established “catch-up” net distribution strategy in the study area was successful in sustaining high coverage with ‘effective nets’ in pregnant women and infants and as would be expected performed poorly in providing ‘effective nets’ to other non-target groups. These findings reinforce the need for universal coverage with sufficient nets distributed to all household members irrespective of age and malaria vulnerability status. Supplementation of the “catch-up” net distribution strategy with universal mass distribution of nets (“keep-up” strategy) at least every two years would sustain high ownership and utilization of ‘effective nets’ to all net users in the study area.

Bed net maintenance behaviour with regard to net washing, retreatment and repair was unsatisfactory in the study community. Even though only a small proportion of nets found in circulation required insecticide re-treatment, the confusion elicited by the survey questions suggests a large knowledge gap with regard to net retreatment. The threat of non-compliance with net retreatment is minimal because nets that require retreatment are slowly being replaced by LLINs. However, frequent washing of bed nets is associated with loss of insecticide [52], and therefore the finding that about a third of the nets were washed more frequently than recommended merits attention. Furthermore, the data showed that frequent washing often leads to poor physical quality of nets and that light coloured nets were frequently washed relative to dark coloured nets. Malaria vector control programmes are encouraged to primarily procure dark coloured bed nets because they are washed less frequently; moreover non-white nets are widely acceptable in Kenya [28]. In contrast to findings from a study conducted in western Kenya where effective net repairing was reported [53], net repairing in this study area was minimal and its impact in improving physical condition of the nets was minor, given that most repaired nets were categorized as ‘ineffective nets’. Panter-Brick and others [48] working in the Gambia detailed that incomplete repairs (not repairing every single hole on the net), poor quality net fabrics, location of holes (repairs were not done if they were located in parts of the net that could stay tucked under the mattresses), cost of repairing (if a tailor is necessary-for large holes) and time constraints as some the obstacles for effective net repairing [48]. To enhance compliance, it is recommended that instructions on maintenance and washing of nets be delivered both orally and in writing at the time of net acquisition, and that this information be subsequently reinforced regularly, preferably by trained community volunteers through locally appropriate instruction [54]. National malaria control programmes should take note of the increasing importance of bed net’s physical integrity and restructure the behaviour change communication (BCC) strategy to highlight bed net maintenance.

This study was conceived and data were collected before the publication of guidelines for monitoring the durability of LLINs under operational conditions [9]. All the same, much of the methodology used to determine net integrity is similar to the one described in these WHO guidelines [9]. Like any other retrospective cross-sectional survey, this study has a number of potential limitations. Firstly, the outcome of whether nets were used and by whom, their age, frequency of washing and the number of users per net were all based on self-reporting, which is subject to respondent bias. Secondly, since this was a cross-sectional survey conducted in an area where nets of different brands are continuously distributed through numerous delivery channels by multiple groups to the study community, it was impractical to estimate the net survivorship and attrition rates. While the aim of the current study was to document all nets that were currently in routine use, it is likely that some nets were missed, and this may affect the findings. Thirdly, it was not possible to assess the contribution of most net brands to the physical deterioration of nets given that most available nets were of the same colour, net fabric, size, and shape (‘olyset’ brand). It is possible that these net attributes could independently influence the physical condition of bed nets in situations where other distribution channels are employed. Fourth, the exact contribution by each demographic group to physical deterioration of nets may have been affected by within-household factors such bed net sharing, alternating bed net user(s) and sleeping space (mattress or mat), and location (bed room, kitchen or sitting room) [55]. Substantial variation in sharing of nets by the different demographic groups was noted in this study (data not shown). Finally, the definition of ‘ineffective nets’ as used here was somewhat arbitrary, and is only supported by few descriptive studies; hence further research that will lead to an operational definition of ineffective nets is needed.

The results of this study have important implications for malaria vector control programmes using ITNs/LLINs. First, even with proper maintenance and repairs, the results suggest that bed nets should be replaced at least bi-annually. Second, while the “catch-up” bed net distribution strategies are sufficient in ensuring adequate coverage with ‘effective nets ‘in the targeted groups, bi-annual mass distribution is necessary to provide similar coverage to the groups not targeted by “catch-up” strategies. Thirdly, malaria control programmes need to put in place monitoring and maintenance strategies that will deliver locally appropriate education messages on net washing and repair.

## Competing interest

The authors declare that they have no competing interests

## Authors’ contributions

FMM, MK, IM, EMM, CHK, and UK designed the study and wrote the manuscript. FMM, MK and PM contributed to the planning, execution and supervision of field activities. FMM, MK and DB performed the statistical analysis. FMM, DB, CHK and UK contributed in data interpretation. All authors contributed equally in preparing the final version of the text and have read and agreed to the manuscript.

## Supplementary Material

Additional file 1 **Additional material S1.** Median PHI and 2nd and 3rd IQ by bednet age in relation to bednet shape and fabric. Click here for file
