# Supplementary material for: Physical condition and maintenance of mosquito bed nets in Kwale County, coastal Kenya
Source: Malar J. 2013 Feb 1;12:46. doi: 10.1186/1475-2875-12-46 (PMC3572415; doi:10.1186/1475-2875-12-46)

Additional material S1: Median PHI and 2^nd^ and 3^rd^ IQ by bednet age in relation to bednet shape and fabric


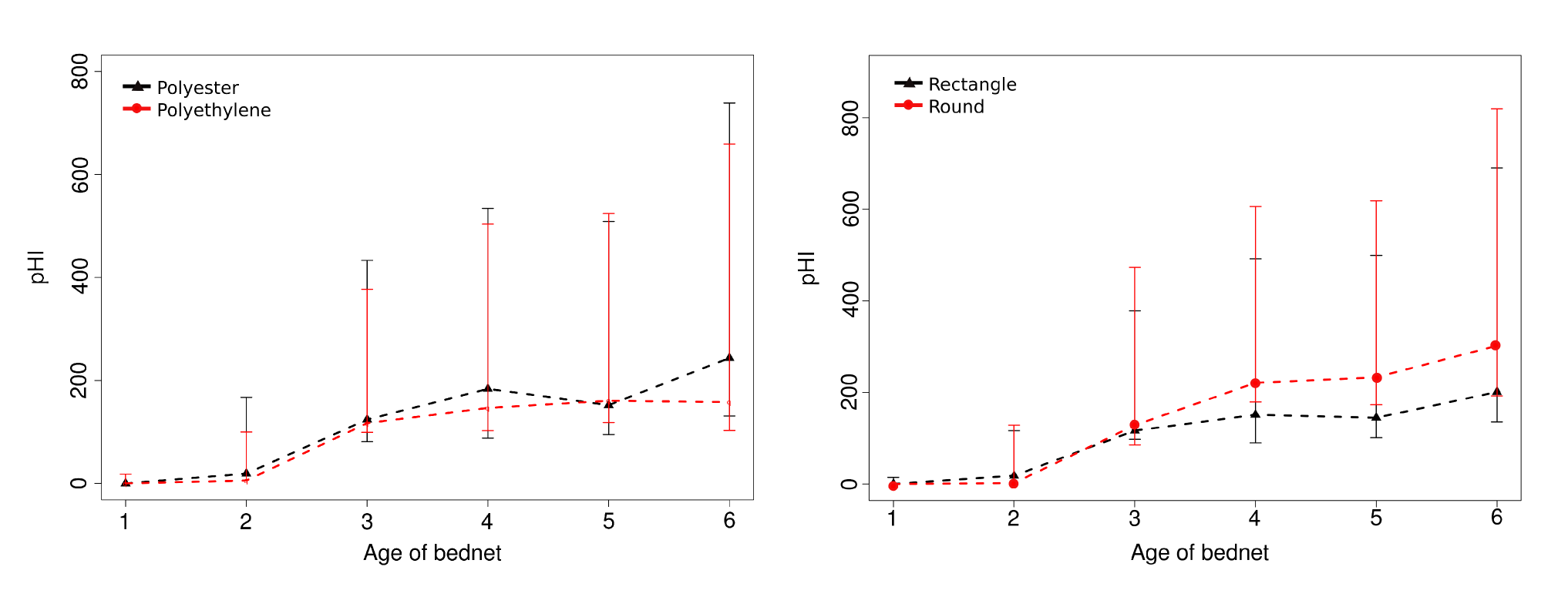

Supplement: Additional file 1 — Additional material S1. Median PHI and 2nd and 3rd IQ by bednet age in relation to bednet shape and fabric. [file 1475-2875-12-46-S1.doc]
